# Performing private database queries in a real-world environment using a quantum protocol

**DOI:** 10.1038/srep05233

**Published:** 2014-06-10

**Authors:** Philip Chan, Itzel Lucio-Martinez, Xiaofan Mo, Christoph Simon, Wolfgang Tittel

**Affiliations:** 1 Institute for Quantum Science and Technology, and Department of Electrical & Computer Engineering, University of Calgary, 2500 University Drive NW, Calgary, Alberta T2N 1N4, Canada; 2 Institute for Quantum Science and Technology, and Department of Physics & Astronomy, University of Calgary, 2500 University Drive NW, Calgary, Alberta T2N 1N4, Canada; 3 Current address: Beijing Institute of Aerospace Control Devices, Quantum Engineering Center, China Aerospace Science and Technology Corporation, Beijing 100854.

## Abstract

In the well-studied cryptographic primitive 1-out-of-*N* oblivious transfer, a user retrieves a single element from a database of size *N* without the database learning which element was retrieved. While it has previously been shown that a secure implementation of 1-out-of-*N* oblivious transfer is impossible against arbitrarily powerful adversaries, recent research has revealed an interesting class of private query protocols based on quantum mechanics in a cheat sensitive model. Specifically, a practical protocol does not need to guarantee that the database provider cannot learn what element was retrieved if doing so carries the risk of detection. The latter is sufficient motivation to keep a database provider honest. However, none of the previously proposed protocols could cope with noisy channels. Here we present a fault-tolerant private query protocol, in which the novel error correction procedure is integral to the security of the protocol. Furthermore, we present a proof-of-concept demonstration of the protocol over a deployed fibre.

Uncertainty in quantum mechanics can be used to provide security in cryptographic applications, allowing quantum cryptographic protocols to relax the typical assumptions required for security (e.g. an adversary with limited computational power), or even avoid them altogether. The use of quantum information has proven extremely successful for key distribution, for which quantum key distribution (QKD)^1^,^2^,^3^ can allow two parties to communicate over a public channel with information theoretic security (i.e. security against an adversary with arbitrarily powerful computational capability, including quantum computers). The application of quantum information theory to other cryptographic tasks is an interesting topic both because of the insight offered into capabilities of quantum versus classical information coding, and because of the possibility of developing new practical cryptographic protocols with improved security. Indeed, there are various proposals and experimental demonstrations of quantum cryptographic primitives such as secret sharing^4^,^5^, coin-flipping^1^,^6^,^7^, bit commitment^8^,^9^, and oblivious transfer (OT)^9^,^10^,^11^,^12^,^13^,^14^,^41^.

When considering cryptographic protocols for deployment, a protocol must ultimately satisfy the following two criteria:

1. Security: The protocol must have a rigorous security analysis based on reasonable assumptions about the adversaries. A strong justification must exist for believing that these assumptions are true.
2. Implementability: The protocol must be implementable with existing technologies, and must function in the presence of loss and noise (which are inevitable in a realistic implementation).

However, initially proposed protocols often do not meet both requirements, and in particular often do not consider loss and/or noise in the quantum channel. Indeed, of the above mentioned protocols, only the bit commitment and OT protocols of ref. 8, 9, 12 are simultaneously loss- and noise-tolerant, and thus are candidates for real-world implementation.

In the case of oblivious transfer, it has been shown that if both parties possess a universal quantum computer it is impossible to simultaneously guarantee that the user, Ursula, can reliably retrieve only a single element while ensuring that the database provider, Dave, has absolutely no knowledge of which element was retrieved^15^. However this does not mean a practical protocol cannot exist. First, note that the security criterion allows for reasonable assumptions about the computational capabilities of the dishonest party (e.g. restricting the adversary from having a universal quantum computer). Indeed, classical OT protocols also rely on one of two assumptions — that at least some fraction of the intermediaries used to perform the query are trustworthy^16^,^17^, or that the adversary has limited classical computational resources^18^. In particular, a quantum protocol has been proposed and implemented based on the assumption that the adversary has limited and noisy quantum storage^9^,^12^,^41^ (which precludes the adversary from possessing a universal quantum computer). However, new developments (e.g. improvements in computational methods^19^,^20^ or in quantum memory^21^,^22^,^23^,^24^,^25^,^26^, respectively) may make these assumptions difficult to justify in the long term. Second, it may be acceptable in practice to relax security conditions of OT — that is, one can allow the user to learn more information from the database, and/or the database may be able to gain some information about the query. Several quantum protocols have been proposed in this vein based on a cheat sensitive model^10^,^11^,^13^,^14^, in which the database provider is kept honest by the possibility of being caught cheating. (This type of security can be sufficient if users wish to purchase information privately from a database who spends significant effort gathering and analyzing data, e.g. to make recommendations to investors, as the database must maintain a high quality of service^13^.) In this setting, the protocol need not prevent the database from gaining any information about the user's query, hence protocols may exist in which the assumptions are easier to justify, or in which no assumptions are required at all. A brief comparison of the properties of the above mentioned protocols for OT and private queries, as well as the protocol we present in this work, is given in Table 1, and we review these protocols in further detail in the Supplementary Information.

In this work, we propose a private query protocol based on the protocols of ref. 13, 14, retaining the advantages of those works while addressing the remaining obstacle to meeting the implementability criterion. This is accomplished using a novel error correction algorithm, in which the algorithm and its associated parameters are tailored to provide the desired level of security in the private query protocol. Furthermore, we note that the novel error correction procedure used to provide fault-tolerance also provides additional opportunities for Ursula to verify Dave's honesty, thus enhancing the cheat sensitive property of the protocol. Hence, we show that error correction is not simply necessary to meet the implementability criterion, but is integral to the security criterion as well.

## Results

As in ref. 13, 14, we implement a cheat sensitive private query protocol based on the SARG04 Quantum Key Distribution (QKD) protocol^27^. The functionality of the protocol can be described as implementing probabilistic *n*-out-of-*N* OT — that is, Ursula will, on average, learn the value of 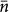 bits (where 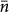 is small) of the database with high confidence (for brevity, we often simply describe such bits as being known to Ursula). She will also have probabilistic knowledge of other bits of the database (i.e. she can guess their value with better than 50% probability). In this scheme, a private query on an *N*-bit database is made possible using an *N*-bit oblivious key (for simplicity, we consider each element of the database to be a single bit) generated by the quantum protocol, in which the goal is to ensure that Ursula knows, on average, 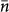 bits of the oblivious key, whose positions are unknown to Dave. In the following sections, we give a description of the protocol for generating an oblivious key and using it to perform private queries, give an overview of the error correction procedure, and then conclude with a brief discussion on security.

### Description of the protocol

**Figure 1:**
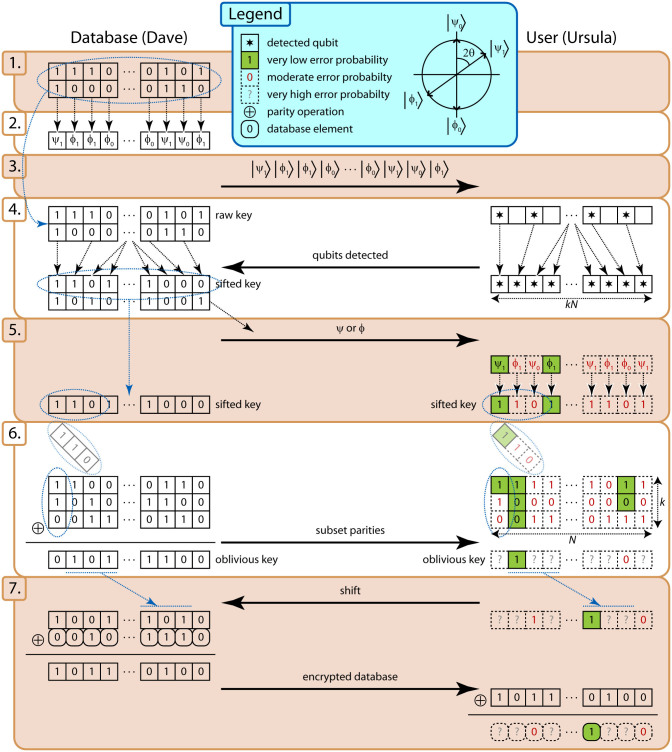
Graphical representation of the private query protocol. The steps indicated on the left margin correspond to the steps described in the text.

A detailed description of the honest protocol for performing a private query is as follows (see Figure 1 for a graphical representation of the protocol):

1. Dave generates two long strings of classical bits uniformly at random, and records their values. Each string should be 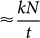 bits in length, where *k* is a parameter determined by the previously agreed-upon error correction procedure (to be discussed later), *N* is the length of the database, and *t* is the transmission of the link between Ursula and Dave.
2. Dave uses each pair of classical bits generated above to choose a quantum state from a set of four previously agreed upon non-orthogonal states (shown in Figure 1), and prepares qubits accordingly. A random bit from the first string determines whether the state is prepared in the 0-basis (spanned by |*ψ*_0_〉 and |*ϕ*_0_〉) or the 1-basis (spanned by |*ψ*_1_〉 and |*ϕ*_1_〉), and the corresponding random bit in the second string determines whether the *ψ* or *ϕ* state in each basis is chosen. The first random string forms Dave's raw key, for which the bit values correspond to the bases in which he prepared the qubits.
3. Dave sends the qubits encoded into single photons to Ursula using a possibly lossy and noisy quantum channel.
4. Ursula makes projection measurements using either the 0- or 1-basis, chosen uniformly at random, and records the measurement bases and the results. Ursula publicly announces the cases in which she detected a photon, and Ursula and Dave both discard all the events in which Ursula failed to detect the photon. The protocol proceeds to the next step once Ursula has succeeded in detecting *kN* photons. Dave keeps the corresponding *kN* bits from his raw key to form his sifted key.
5. Dave publicly announces his second string of random bits (used to select whether he encoded the qubits into a *ψ* or *ϕ* state), which, combined with knowledge from Ursula's measurements (and, for the moment, assuming a noiseless channel), allows her to conclusively identify whether the state was encoded in the 0- or 1-basis with probability 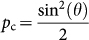. Note that when Ursula's measurements yielded inconclusive results, which occurs with probability *p*_i_ = 1 − *p*_c_, she gains probabilistic information about the basis. This information can be quantified by the probability that she incorrectly identifies the basis, 
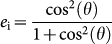
. A noisy channel will affect the probabilities *p*_c_, *p*_i_, and *e*_i_, as well as result in a non-zero error rate for conclusive measurements, denoted *e*_c_. Like Dave, Ursula associates classical bit values to the quantum states based on the basis, and forms her sifted key using the most likely values of the bits given her measurement results.
6. Dave divides his sifted key into *N* *k*-bit blocks, and computes each bit of his oblivious key as the parity of the *k* bits in each block (the parity is 0 if an even number of the *k* bits is 1, and 1 otherwise). He publicly announces which bits form each block. In addition, according to a previously agreed upon error-correcting code, he also sends the parities of several subsets of the *k* bits to Ursula. Using this information, along with her sifted key and knowledge of whether the measurements were conclusive or inconclusive, Ursula computes the most likely value of each oblivious key bit, as well as the probability that this value is incorrect, denoted *e*_k_. The error-correcting code is selected such that Ursula will only have a high confidence (or low *e*_k_) in 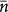 bits on average, where 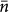 is typically a few bits. If Ursula does not learn any bits of the protocol (due to its probabilistic nature), the protocol must be restarted.
7. Ursula selects a shift value that aligns one of the bits she knows in the oblivious key to the bit in the database that she wants to know. She communicates this shift value classically to Dave, who applies the shift to his oblivious key, and then uses it to encrypt the database using the one-time-pad^28^. He then sends the encrypted database to Ursula, who can only decrypt the bits for which she knows the corresponding oblivious key bit. If Ursula knows multiple bits of the oblivious key she will learn multiple bits of the database. However, the shift only allows her to select the location of a single bit of the database, with the remaining learned bits distributed randomly.

### Error-correcting codes for private queries

Let us now examine step 6 of the protocol in more detail. Our error correction procedure (see Supplementary Information for a full description) is inspired by syndrome decoding of error-correcting codes such as Hamming codes^29^, which can operate on a few bits at a time. However, it is important to note that in the context of private queries error correction is integral to determining how much information Ursula learns about the oblivious key, creating unique requirements that made it necessary to investigate and design novel error-correcting codes and error correction procedures. In particular, the goal when designing an error-correcting code for private queries is not to simply maximize the probability of successful decoding as it is in standard communications applications. Rather, a specific success probability is desired in order to ensure that Ursula only learns a few bits of the oblivious key. Furthermore, to prevent Ursula from learning a large amount of probabilistic information about the remaining bits of the key, it is desirable to keep *e*_k_ as high as possible in those cases in which decoding does not succeed.

In addition there are two main technical differences between error correction in private queries and in communications. First, note that in order to recover the value of the oblivious key bit, Ursula need only determine the parity of the *k*-bits, and not the individual values of the *k* bits as would typically be the case for error correction. Hence, the error correction procedure seeks the most likely parity of the *k*-bit block, and successful decoding does not depend on having a high probability of identifying the correct values of the *k*-bit block as long as it is possible to identify whether an even or odd number of errors occurred. Second, the input bits can be divided into those with low error rate (conclusive measurements), and those with very high error rate (inconclusive measurements). We note that it is the interaction of this latter property with the short block lengths used (*k* ≤ 10) that allows uncertainty to be maintained after error correction, thereby limiting the amount of information that Ursula learns about the database.

The error-correcting codes used in this work are tailored based on the experimental parameters (i.e. conclusive and inconclusive probabilities, *p*_c_ and *p*_i_ and the associated error rates *e*_c_ and *e*_i_) in order to achieve the goals discussed above. In order to quickly evaluate error-correcting codes, we define two thresholds, *t*_U_ and *t*_D_. When *e*_k_ ≤ *t*_U_, Ursula considers the oblivious key bit to be known. When *e*_k_ ≤ *t*_D_, Dave considers Ursula to have significant partial information about that bit. These thresholds should be selected based on the requirements of the application. In this work, we use *t*_U_ = 10^−3^ and 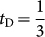. In order to reduce the probability of error in Ursula's oblivious key bit below her threshold (i.e. *e*_k_ ≤ *t*_U_), the error correction process must sufficiently reduce *e*_k_ when her quantum measurements succeeded in obtaining a large amount of information about the *k* bits (i.e. when most or all measurements were conclusive). However, the error correction will also reduce *e*_k_ if several measurements were inconclusive. Hence, the error rate for inconclusive measurements, *e*_i_, is of particular importance to the fraction of bits for which *e*_k_ ≤ *t*_D_. With this in mind, a smaller angle between states (characterized by *θ* as shown in Figure 1) has, in addition to those benefits noted in ref. 14 (i.e. reduced quantum communication, improved database security, and better control over the number of bits Ursula learns), the benefit of reducing the partial information from inconclusive measurements. However, there is a trade-off between these benefits and the fact that the error rate for conclusive measurements is also increased due to a reduced signal-to-noise ratio, making it more difficult to achieve *e*_k_ ≤ *t*_U_. A detailed description of the selection of our error-correcting codes is given in the Supplementary Information.

### Security of the protocol

Let us now discuss how the steps in the above protocol contribute to security, beginning with a discussion of user privacy. User privacy is protected by the cheat sensitive property of the protocol, which allows a dishonest database to be detected. This property stems from step 4 of the protocol as Ursula randomly selects between two possible (non-commuting) measurements and does not announce which measurement she performed. Her security thus stems from the complementarity principle as her interpretation of her measurement results is dependent on her choice of measurement basis, with the protocol designed such that the classical bit value she assigns to each result is perfectly correlated with her basis choice (see step 5 and the Supplementary Information for more details). In the case that Dave is honest (and for the moment, assuming a noiseless system), Ursula's classical bit values for conclusive measurements will also be perfectly correlated with the classical bit values Dave used to select which quantum states he encodes. If Dave is dishonest, and supposing he can send a state such that Ursula's measurement is conclusive regardless of which measurement basis she chooses (a realistic attack is analyzed in the Supplementary Information), Ursula's interpretation of her measurements remain unchanged, hence her classical bit values are still perfectly correlated to her choice of basis. Since this choice is never revealed to Dave, he does not know which bit value she obtains. This leads to the cheat sensitivity in the protocol, as the dishonest database may be detected during error correction (since he sends parity values uncorrelated with Ursula's classical bit values), or after completion of the protocol since he may send incorrect query results. Furthermore, note that the error correction procedure in step 6 only involves one-way communication from Dave to Ursula, hence Dave gains no information regarding the results of the error correction procedure.

On the other hand, Ursula's limited knowledge about the oblivious key stems from the superposition principle in quantum mechanics. Specifically, note that in step 2 Dave prepares qubits in non-orthogonal states, and that Ursula can thus not deterministically distinguish between these states. As such, Ursula's measurements only give her limited information, even after Dave reveals some information about which state he sent in step 5. Furthermore, note that Ursula must declare which bits were lost during transmission (or detection) in step 4, prior to receiving classical information indicating whether a *ψ* or *ϕ* state was sent. This makes the protocol loss-tolerant while ensuring that Ursula cannot choose which bits to keep based on whether her measurements were conclusive or inconclusive, even if she uses a heralded quantum memory to delay her measurements until after step 5. Note that in step 6, Ursula does have the ability to restart the protocol if the results are unfavorable as Dave cannot verify whether she indeed learned no bits of the oblivious key. However, choosing an error-correcting code such that 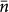 is a few bits ensures that the probability for Ursula to not know any bits is very low, and allows Dave to abort the protocol after a small number of declared failures by Ursula (preventing her from repeatedly declaring failure until she obtains a very favorable result).

Furthermore, a dishonest user may gain an advantage by deviating from the honest protocol. It has been shown that Ursula could perform an unambiguous state discrimination (USD) measurement^30^,^31^ in order to slightly improve her probability of conclusive measurements, which allows her to learn a few additional bits of the oblivious key^13^. However, this comes at the expense of gaining no information about the bit value (i.e. *e*_i_ = 0.5) when the USD measurement gives inconclusive results. While this probabilistic information was not previously considered useful^13^,^14^, it is an important input to the error correction process. Thus, the effectiveness of this attack is reduced in the presence of error correction, and our analysis in the Supplementary Information shows that in some cases performing a USD measurement actually reduces the number of bits of the oblivious key that Ursula learns as compared to the honest measurements. Note that only individual USD measurements have been considered, and coherent attacks (e.g. an optimized USD measurement on the *k* qubits that form each oblivious key bit) remain an interesting open question.

We also note that Ursula and Dave are adversarial in nature in the protocol, and thus may not cooperate when estimating the error rate in order to select an appropriate error-correcting code. An error-correcting code that is not well suited to the actual error rate in the system will either result in Ursula learning too few or too many bits of the oblivious key, but does not impact user security. Hence the database does not have any motivation to falsify the error rate, but the user would like the database to think the error rate is larger than it is in reality, leading to the selection of an error-correcting code that gives her more information. In our analysis (detailed in the Supplementary Information), we find that Dave can ensure that he has a reasonable level of security by determining the error rate of devices under his control (potentially by intentionally introducing noise) and selecting an error-correcting code accordingly. In addition, even if Ursula's devices introduce some additional error that Dave does not account for in his security analysis, the protocol is still successful for her.

### Experimental and simulated performance of our protocol

**Figure 2:**
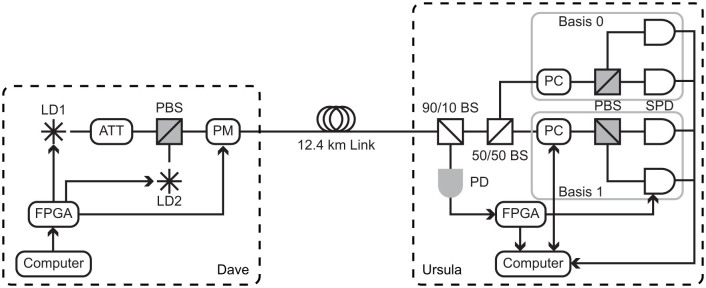
Diagram of the experimental setup. The database (Dave) uses a computer and field-programmable gate-array (FPGA) to control the generation of polarization qubits via an attenuated laser diode (LD1 and ATT) and polarization modular (PM). Quantum frames^32^ (sequences of strong light for timing and stabilization) are generated by a second laser diode (LD2) and merged using a polarizing beam-splitter (PBS). Light is transmitted from Dave to Ursula through a 12.4 km dark fiber link with 4.5 dB loss between SAIT Polytechnic and the University of Calgary. Ursula splits off 10% of the incoming light (90/10 BS) to a photodiode (PD) used to detect the quantum frames. The 50/50 BS is used to passively select a random measurement basis. The apparatus for each basis consists of a polarization controller (PC), a PBS, and two single photon detectors (SPD) to make the projection measurement. Upon detecting a quantum frame, Ursula's FPGA triggers the SPDs and initiates data collection by the computer, or polarization compensation, as appropriate.

We performed an experimental demonstration of private queries over a 12.4 km fiber link between the University of Calgary and SAIT Polytechnic, using our BB84^1^ QKD system^32^ (with a small modification to the hardware to set *θ* = 35.6° ± 0.49° — all other differences between our protocol and BB84 QKD are in the classical post-processing). Our experimental setup is shown in Figure 2 (see ref. 32 for a detailed description). Note that our demonstration uses weak coherent pulses rather than single photons, and hence database privacy requires the assumption that Ursula is not able to exploit pulses containing multiple photons (adapting the protocol for weak coherent pulses, e.g. using decoy states as in QKD^33^,^34^,^35^,^36^, remains an open question, and we discuss this possibility further in the Supplementary Information). We consider a database size of *N* = 10^6^ and, based on measured error rates for our system, an error-correcting code with *k* = 10 was selected, thus requiring 10^7^ measured qubits per query. Note that we did not consider *k* > 10 due to computational constraints when searching for the best possible construction of the error-correcting code. A total of 11 queries was performed using a mean number of photons per pulse of *µ* = 0.95 ± 0.047 to show that the protocol can function at the single photon level. In this setting, our system took approximately 4.5 hours to accumulate the 10^7^ bits of data needed for one private query. In order to quickly collect statistics, we repeated the experiment with mean number of photons per pulse increased to *µ* = 9.5 ± 0.47, performing 104 queries. While the multi-photon emissions at this *µ* are likely to compromise the security of the protocol if Ursula monitors the pulses outside Dave's laboratory, this value corresponds to ~ 0.95 photons per pulse at the detectors, ensuring that multi-photon detection events do not skew the detection statistics. The measured parameters that determine the performance of the protocol are shown in Table 2 (note that the experimentally measured parameters at both mean photon numbers are the same to within one standard deviation), along with parameters for a theoretical simulation of what could be achieved using state-of-the-art detectors^37^,^38^. These detectors allow for significantly reduced noise (they feature dark count rates ≈ 100 Hz), and, in the case of ref. 37, detection efficiencies up to 93%. With the improved signal-to-noise ratio, we select the parameters of the protocol to be *θ* = 25° and *k* = 9.

**Figure 3:**
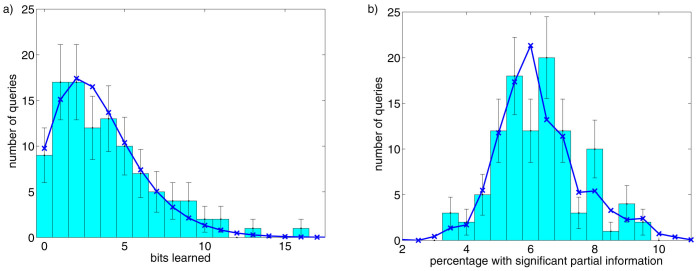
Histograms for the information gained by the user in the 104 queries performed in the *µ* = 9.5 ± 0.47 case. (a) The number of bits learned by the user. (b) The percentage of the database of which the user learns significant partial information. In both figures error bars for the experimental results represent one standard deviation assuming Poissonian counting statistics, and the blue crosses show the expected distribution obtained from Monte Carlo simulations.

The experimental and simulated results for these codes are shown in Table 3. The simulated results corresponding to our experiment are derived from Monte Carlo simulations taking into account the variation in the parameters shown in Table 2. Figure 3 compares the distribution of the results over the 104 queries performed in the *µ* = 9.5 ± 0.47 case with the simulation results, showing good agreement between the two. Note that in both experimental cases, no errors were observed in the bits learned by Ursula (i.e. for which *e*_k_ ≤ 10^−3^), with a total of 45 bits learned in 11 queries when *µ* = 0.95 ± 0.047 and 405 bits learned in 104 queries when *µ* = 9.5 ± 0.47.

In addition, our simulation results show that the primary obstacle to improving database security in the protocol is noise in the system, which can be greatly reduced by state-of-the-art single photon detectors. These detectors can also improve the rate at which queries can be performed by almost an order of magnitude because of their higher detection efficiencies. Further improvement of this rate is straightforward, as QKD systems can easily be adapted to perform this protocol. A state-of-the-art BB84 QKD system has shown that data can be accumulated at a rate of 10^6^ to 10^7^ bits per second, depending on the distance between Ursula and Dave^39^. For the parameters in our experimental demonstration, this would allow one private query to be performed every few seconds. The amount of data required can also be reduced by repeating a short oblivious key over a longer database and then applying a shift as before to allow Ursula to select the desired bit. This would allow queries to be performed more often, or equivalently, allow queries to be performed on a larger database in the same amount of time. However, this comes at the expense of database security, as the user is able to learn additional bits for each repetition of the key (though not in locations of her choice, as only a single shift value is communicated). We also note that a modification to the protocol of ref. 13 has recently been proposed that reduces the amount of quantum communication required^40^, however applying this modification to our protocol is not straightforward.

## Discussion

We have proposed and demonstrated, over deployed optical fibres, a quantum protocol for private queries using the cheat sensitive model. This first demonstration of private queries in a real-world setting was made possible by the development of a protocol which integrates a novel error correction procedure. Our analysis of this protocol has shown that error correction plays a pivotal role in the security, both in terms of controlling how much information the user learns, and in providing the ability for Ursula to detect a dishonest database. While our security analysis is currently limited to several specific attacks, it is important to note that the error correction should be viewed as an important tool for tailoring the amount of information learned by the user, and hence may be adaptable to a more general scenario where Ursula makes more powerful measurements. In this general view, database security stems from the fact that quantum mechanics allows a protocol to be designed where the user cannot extract full information about the quantum states sent, and error correction allows the extracted information to be processed into an oblivious key with the desired distribution of information for private queries. Furthermore, quantum mechanics allows such a private query protocol to be set up such that the correlation between Ursula and Dave's classical raw key bits is destroyed if Dave can control which bits of the oblivious key Ursula learns. Hence, the methods presented in this work should provide a strong basis for the further development of cheat sensitive quantum protocols.

## Supplementary Material

Supplementary Information Supplementary Information: Performing private database queries in a real-world environment using a quantum protocol

## Figures and Tables

**Table 1:** Comparison of the ability of various protocols for private queries to meet the two criteria for deployment (security and implementability). Note that the cheat sensitive security model may offer the possibility for security with no additional conditions since the impossibility proof^15^ may not apply

		Security		Implementability	
	protocol	security model	conditions for which security is known to hold	loss-tolerant	fault-tolerant
classical information	computational^18^	standard	adversary has limited classical and quantum computational capability	N/A	N/A
	trusted^16^,^17^	standard	trusted intermediaries are available	N/A	N/A
quantum information	noisy-storage^9^,^12^,^41^	standard	parameters of the adversary's quantum memory (e.g. decoherence as a function of time) are known	yes	yes
	GLM^10^	cheat sensitive	no additional conditions	no	no
	QKD based^13^,^14^	cheat sensitive	specific attacks discussed in refs. 13, 14	yes	no
	our protocol	cheat sensitive	specific attacks discussed in this work	yes	yes

**Table 2:** Parameters for the private query protocol as measured in our experiment with standard detectors, and simulated for low-noise detectors. The value of *θ* (including standard deviation) is measured using classical light. For the probabilities of conclusive measurements, *p*_c_, and error rates for conclusive and inconclusive measurements, *e*_c_ and *e*_i_, the standard error expected based on Poissonian counting statistics for the 10^7^ bits in each query is negligible compared to the observed variations across the queries performed. The observed standard deviations are attributed to time-varying error in the alignment of the measurement bases at the receiver as a result of channel instability. Note that the measurement results for the *µ* = 9.5 ± 0.47 case show more variation in the parameters than for the *µ* = 0.95 ± 0.047 case due to short-term fluctuations that are averaged out by the long data collection time needed to acquire the 10^7^ bits per query in the *µ* = 0.95 ± 0.47 case

	standard detectors		low-noise detectors
*µ* (photons)	0.95 ± 0.047	9.5 ± 0.47	1
*θ* (°)	35.6 ± 0.49	35.6 ± 0.49	25
*p*_c_ (%)	16.1 ± 0.29	16.1 ± 0.93	9.22
*e*_c_ (%)	4.4 ± 0.59	4.6 ± 0.38	1.91
*e*_i_ (%)	41.24 ± 0.08	41.3 ± 0.64	45.12
*k* (bits)	10	10	9

**Table 3:** Experimental and simulated results for the quantum private queries. The following figures of merit are used: the average number of bits learned by the user per query, 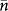, the average proportion of the database where the user has significant partial information (i.e. *e*_k_ ≤ *t_D_*), 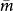, and the failure probability (i.e. that the user learns zero bits), *P*_0_

	*µ* = 0.95 ± 0.047		*µ* = 9.5 ± 0.47		low-noise
	experimental	simulated	experimental	simulated	simulated
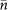 (bits)	4.1 ± 2.4	3.2 ± 1.1	3.9 ± 3.1	3.5 ± 1.9	4.35
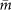 (%)	6.1 ± 0.25	6.1 ± 0.25	6.3 ± 1.4	6.3 ± 1.3	0.96
*P*_0_ (%)	9.1 ± 9.1	8.8	8.7 ± 2.9	9.4	1.29
