# Supplementary material for: Performing private database queries in a real-world environment using a quantum protocol
Source: Sci Rep. 2014 Jun 10;4:5233. doi: 10.1038/srep05233 (PMC5381472; doi:10.1038/srep05233)
Supplement: Supplementary Information — Performing private database queries in a real-world environment using a quantum protocol [file srep05233-s1.pdf]

Supplementary Information: Performing private database queries in a real-world environment using a quantum protocol

Philip Chan,[1] Itzel Lucio-Martinez,[2] Xiaofan Mo†,[2] Christoph Simon,[2] and Wolfgang Tittel[2]

[1]*Institute for Quantum Science and Technology, and Department of Electrical & Computer Engineering, University of Calgary, Canada*

[2]*Institute for Quantum Science and Technology, and Department of Physics & Astronomy, University of Calgary, Canada*

†*Current address: Beijing Institute of Aerospace Control Devices, Quantum Engineering Center, China Aerospace Science and Technology Corporation, Beijing 100854.*

I. REVIEW OF OBLIVIOUS TRANSFER AND PRIVATE QUERIES

An ideal 1-out-of-N oblivious transfer protocol simultaneously guarantees that (a) that the user, Ursula, is able to retrieve a single element from the N-bit database, and (b) that the database provider, Dave, cannot gain any information about which element was retrieved. However, it has been shown that, assuming a universal quantum computer, if a protocol meets condition (b) then condition (a) implies that Ursula can access every element of the database[1]. As such, it is impossible for a protocol to implement ideal oblivious transfer without making assumptions in the security model. Alternatively, the class of protocols we refer to as private queries avoids the impossibility proof by implementing functionality similar to 1-out-of-N OT. Such protocols offer a reduced level of privacy up front, but this reduction in privacy may allow secure protocols using assumptions that are easier to justify, or in which no assumptions are required at all. In this section, we briefly review protocols for oblivious transfer and private queries.[1]

In classical information theory, protocols for OT rely on one of two assumptions — that at least some fraction of the intermediaries used to perform the query are trustworthy[2, 3], or that the adversary has limited classical computational resources[4]. The former assumption can be difficult to assess, as one must both believe that the intermediaries will not collude with each other, and that their infrastructure is secured against attacks. The latter assumption is shared with today's public key cryptography infrastructure, and is hence well justified in the short term. However, in the long term, the security of such systems can be compromised by advances in algorithms (e.g. ref. 5) or hardware such as a quantum computer[6].

A quantum 1-out-of-2 OT protocol has also recently been proposed using the noisy-storage model[7], where it is assumed that the dishonest party has a limited ability to store quantum information, and that the amount of information that can be faithfully stored decreases over time due to noise in the quantum memories (note that this protocol is loss- and fault-tolerant, as quantum memories are not required by the honest protocol). Since quantum memories are a basic component in a universal quantum computer, this assumption means that the proof that ideal OT is not possible[1] does not apply. Thus, perfect privacy is possible under this model, and this has indeed been shown in the protocol of ref. 7, 8. An experimental demonstration of the protocol in [8] has also recently been performed[9], showing that it meets the implementability criterion. As with the classical OT protocols relying on assumptions about the adversaries computational capabilities, this assumption is well justified in the short term given current quantum memories. However, there is no fundamental principle limiting the adversaries ability to store quantum information, and recent advances in quantum memories[10–15] threaten the validity of this assumption in the long term.

The private queries approach to OT using cheat sensitivity was first proposed in ref. 16. This protocol does not satisfy condition (b) above, since a dishonest database could gain complete information about which element Ursula retrieved. However, the protocol still offers security for Ursula as she has, in principle, the potential to detect Dave's attempt to gain information about her query, thus discouraging Dave from cheating. Note that condition (a) was also not satisfied, as a dishonest user could sacrifice her ability to verify Dave's honesty in order to obtain a small number of additional elements (although, this is not a significant loss of privacy for the database if N is large). An experimental proof-of-principle demonstration of this protocol was subsequently performed[17], however, as Dave could hide his attempts to cheat if there was significant transmission loss and/or errors in the quantum channel, the protocol is not practical under realistic conditions. Ref. 18 then proposed a probabilistic n-out-of-N OT protocol based on the SARG04 Quantum Key Distribution (QKD) protocol[19], which was then generalized[20]. This protocol allows Dave to gain information about Ursula's query, but only at the risk of introducing errors into the element Ursula retrieved, thereby allowing a dishonest database to be detected. The protocol also did not satisfy condition (a) above as Ursula gains probabilistic information about elements of the database she does not request. Interesting features of this protocol are the ability to tolerate loss in the channel, as well as the fact that it is simple to implement using existing QKD technology. However, noisy channels were left as an open question, preventing implementation

of the protocol in realistic scenarios. Finally, our protocol proposed in this work represents the first cheat sensitive protocol to be both loss- and fault-tolerant, making it suitable for implementation in a realistic environment.

II. QUANTUM STATE IDENTIFICATION

In our protocol, the database provider, Dave, encodes each qubit into one of four randomly chosen quantum states, $|\psi_0\rangle$, $|\psi_1\rangle$, $|\phi_0\rangle$ or $|\phi_1\rangle$, as shown in Figure 1. The user, Ursula, measures each qubit in either the 0-basis, spanned by $|\psi_0\rangle$ and $|\phi_0\rangle$, or the 1-basis, spanned by $|\psi_1\rangle$ and $|\phi_1\rangle$. After these measurements, Dave tells Ursula whether each qubit was encoded into one of the ψ states or one of the ϕ states. In order to demonstrate the state identification process, suppose Ursula measured in the 0-basis, and Dave declares that he sent one of the ψ states. If Ursula's measurement result was $|\phi_0\rangle$, she knows Dave could not have sent $|\psi_0\rangle$ as these two states are orthogonal. Hence Dave must have sent $|\psi_1\rangle$. This is a conclusive result, and occurs with probability $p_c = \frac{\sin^2(\theta)}{2}$. Alternatively, if Ursula's measurement result was $|\psi_0\rangle$, she only knows that the state was more likely to have been $|\psi_0\rangle$ than $|\psi_1\rangle$. This is an inconclusive result, occurring with probability $p_i = 1 - p_c$. As the two potential states are associated with different classical bit values (as indicated by the subscripts), Ursula only gains probabilistic knowledge from this measurement result. This corresponds to an error rate of $e_i = \frac{\cos^2(\theta)}{1+\cos^2(\theta)}$ in the ideal case (i.e. when no other sources of error are present).

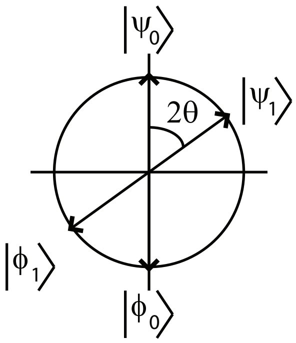

Supplementary Fig. 1: Quantum states used in the private query protocol shown on a plane of the Bloch sphere.

Let us now examine how this state identification process leads to user privacy, considering first the honest protocol. In the above example where Dave sent one of the ψ states and Ursula measures in the 0-basis, note that Ursula can only get a conclusive measurement result if Dave sent the $|\psi_1\rangle$ state. If Ursula instead measures in the 1-basis, she can only get a conclusive measurement if Dave sent the $|\psi_0\rangle$ state. Hence, for any given qubit that Dave sends, Ursula's choice of measurement determines whether a conclusive result is possible — she never gets a conclusive result if she measures in the same basis in which Dave encoded the qubit. Since she never reveals her choice of measurement basis to Dave, he cannot know which of her measurements gave conclusive results.

Now, let us consider the case in which Dave is dishonest. In this case, Dave wishes to break the correlation between Ursula's choice of measurement basis and the conclusiveness of her measurement results. Ideally, he would like to choose whether Ursula will get a conclusive or inconclusive measurement result, regardless of which measurement she makes. For ease of discussion, we assume here that Dave can send a quantum state that accomplishes this goal (we discuss a more realistic attack in Section V). Since Ursula is honest, she makes the same measurements as before, and interprets them assuming Dave is honest. In the above example, in which Dave declares he sent one of the ψ states, if Ursula measures in the 0-basis, she will either conclusively identify that Dave sent the $|\psi_1\rangle$ state, or inconclusively identify that Dave likely sent the $|\psi_0\rangle$ state. If she instead measured in the 1-basis, she will either conclusively identify that Dave sent the $|\psi_0\rangle$ state, or inconclusively identify that Dave likely sent the $|\psi_1\rangle$ state. Recall that the classical bit values that form the raw keys in the protocol are given by the basis of the state that Ursula believes Dave sent (and correspond to the subscripts in the ket notation). Thus, Ursula's raw key bits are anti-correlated with her choice of measurement basis for conclusive results, and correlated for inconclusive results. Hence, if Ursula's choice of measurement basis does not determine whether a measurement is conclusive, it instead determines her raw key bits. In this case, since she never reveals her choice of measurement basis, Dave cannot know her raw key bits. This leads to the cheat sensitivity in the protocol as the fact that Dave has no knowledge of Ursula's raw key bits may be detected during error correction, and if not detected, results in incorrect query responses. A more detailed analysis of the cheat sensitivity is given in Section V.

III. ERROR CORRECTION

We use a parity-based forward error-correcting code operating on k-bit blocks (corresponding to the k bits used to compute one oblivious key bit), where Dave sends the parity of several subsets of the k bits to Ursula. The construction of the code is normally described as a parity check matrix, denoted $\mathbf{H}$, and is known to both Ursula and Dave. The parity computation for the jth oblivious key bit is then given by:

$$\vec{p}_j = \mathbf{H}\vec{d}_j \pmod 2 \tag{1}$$

where $\vec{p}_j$ is a vector of computed parity bits (which Dave sends to Ursula) and $\vec{d}_j$ is a vector containing the k bits that Dave uses to compute a single oblivious key bit. For each oblivious key bit, Ursula has a corresponding k-bit vector, $\vec{u}_j$, in which each bit stems from a conclusive or an inconclusive measurement that have, respectively, error rates of e_{c} and e_{i}. Ursula can estimate these error rates over the entire protocol by comparing the parities, $\vec{p}_j$, she receives from Dave and the parities she computes locally using $\vec{u}_j$. Using these error rates, Ursula's error correction procedure for each oblivious key bit is as follows:

1. Rule out those combinations of values for the k bits that are not consistent with the values for $\vec{p}_j$ received from Dave.
2. Divide the remaining possibilities into two sets — those that correspond to an oblivious key bit of 0, and of 1.
3. Based on the measurement results and estimated error rates, calculate the probability that each combination of values for the k bits is correct. The set with the higher total probability determines the most likely value of the oblivious key bit.
4. Compute the probability of error in the oblivious key bit, e_{k}.

Note that Ursula can significantly reduce the computation required for error correction by performing this procedure only if almost all of the k bits were measured conclusively. In doing so, she only performs error correction if there is a possibility that the result will satisfy $e_{\text{k}} \leq t_{\text{U}}$.

The error correcting codes used in this work are given by:

$$\mathbf{H}_{35.6} = \begin{bmatrix} 1&0&0&0&0&1&0&0&0&0 \\ 0&1&0&0&0&1&1&1&0&0 \\ 0&0&1&0&0&1&1&0&1&0 \\ 0&0&0&1&0&1&0&1&1&0 \\ 0&0&0&0&1&1&0&0&0&1 \end{bmatrix} \tag{2}$$

for $\theta = 35.6°$ and

$$\mathbf{H}_{25} = \begin{bmatrix} 1&0&0&0&0&1&0&0&0 \\ 0&1&0&0&0&1&0&0&0 \\ 0&0&1&0&0&1&1&1&0 \\ 0&0&0&1&0&1&1&0&1 \\ 0&0&0&0&1&1&0&1&1 \end{bmatrix} \tag{3}$$

for $\theta = 25°$. They were selected using an exhaustive search of potential error-correcting codes for $k \leq 10$. The probability distribution for e_{k} is computed for each code based on the parameters in Table 2 of the main text, and the selected codes provide a low probability for $e_{\text{k}} \leq t_{\text{D}}$ (indicating a small amount of information leakage to Ursula) as well as a suitable probability for $e_{\text{k}} \leq t_{\text{U}}$ (ensuring that Ursula learns a few bits of the oblivious key on average). Note that both matrices are in reduced row echelon form (i.e. no 1's appear below the leftmost 1 in any row). This is due to the fact that the possible k-bit vectors remaining after step 1 of the error correction process (i.e. consistent with the parity information received from Dave) are given by the possible solutions of the system of linear equations in Eq. 1, hence any error correction codes that have the same reduced row echelon form behave identically in the error correction process. The search space was thus limited by only considering matrices in reduced row echelon form.

IV. REQUIREMENTS FOR SECURITY

The security of the experimental results presented in Table 3 and Figure 3 of the main text hold given that the dishonest party is limited to non-quantum attacks (e.g. an arbitrarily powerful classical computer, which would be sufficient to break computational protocols using classical information such as [4]). Furthermore, results for the security of the protocol against several quantum attacks are presented in the Section V. Note that these limitations on the attacks a dishonest party can perform are a result of the current security analysis of the protocol, and may not be required in general. It remains an open question as to what limitations on the dishonest party, if any, are required to achieve a sufficient level of security. Based on the attacks we have studied, we believe that at a fundamental level, the security of the protocol stems from the complementarity principle (protecting the user's security) and the superposition principle (protecting the database's security). In addition, we note that the error-correcting code in our protocol can be selected in order to provide less information to Ursula in order to compensate for an increased information gain from more powerful quantum measurements. Thus, it may be possible to adopt such measurements as the legitimate procedure for the user, provided that the measurements are feasible technologically.

We also note that the security results are valid only if certain requirements are met. These requirements are listed below, beginning with those that are required in general, followed by those that are imposed by the current security analysis:

1. Ursula's and Dave's laboratories are secure (i.e. no information leaves their laboratories except as specified in the protocol). (Required for any protocol.)

2. Quantum theory is correct and complete. (Required for any quantum protocol.)

3. The dishonest party is limited to the attacks covered in the current security analysis (see Section V).

4. In our experimental demonstration, it is also necessary to assume that the user is not able to take advantage of multi-photon pulses that result from using a source of weak coherent pulses. While this assumption can be avoided if Dave uses a single photon source, the implementation of weak coherent pulses is much simpler from a technological perspective. Thus, it is desirable for the protocol to be secure for weak coherent pulses without the need for additional assumptions. The decoy state techniques used in QKD [21–23] provide security against an adversary capable of exploiting multi-photon pulses. However, these techniques cannot be directly applied in cases where the two parties are adversarial, as is the case in private queries, and must be modified to account for the fact that the two parties need not be honest in the protocol [24]. However, it is not clear that the techniques in ref. 24 can be applied directly to our protocol. In particular, Ursula may gain an advantage by manipulating the aggregate statistics of the decoy state protocol by conducting an attack (e.g. by lying about detections) during a subset of the protocol while acting honestly for the remaining subset. Analyzing and adapting decoy state techniques for our protocol is thus an interesting open question. It may also be possible for Dave to base his estimate of the additional information that may have been extracted from multi-photon pulses on a characterization of his source. Regardless of how Dave quantifies Ursula's information gain due to multi-photon pulses it can be accounted for by selecting an appropriate error-correcting code. If the information gain is sufficiently small, the protocol can provide a suitable level of database security while maintaining a high success probability for the user.

V. CHEATING STRATEGIES

In this section we discuss the attacks on individual qubits proposed in [18, 20]. The discussion below shows that the error correction step provides improved security for the protocol against these individual attacks. Optimization of error correction in view of coherent attacks remains an interesting open question, as does an analysis of fully general quantum attacks and an information theoretic treatment of our protocol. Furthermore, we comment on the issue of error rate estimation between adversarial parties. As example cases for these discussions, we consider the mean parameters (θ, p_{c}, e_{c}, and e_{i}) measured with $\mu = 0.95 \pm 0.47$ using standard detectors and the simulated parameters for low-noise detectors (see Table 2 in the main text). For the measured parameters, we do not consider the observed variances since they are specific to the system used to implement the honest protocol.

A. User Privacy

Let us first consider an attempt by the database to determine which piece of information Ursula is interested in. Recall that our protocol does not prevent a dishonest database from gaining some information about Ursula's query, but is cheat sensitive in that it gives Ursula the possibility of detecting such an attack. Performing the attack described below does not require any additional technology, as it simply requires Dave to send quantum states that either maximize or minimize the probability, p_{c}, that Ursula will believe her measurement was conclusive [18]. In order to determine Ursula's query, Dave seeks to have Ursula learn only a single bit of the oblivious key whose position is known to him, thus he maximizes p_{c} for the k bits that form one oblivious key bit in an attempt to convince Ursula that she knows a particular bit of the oblivious key. He then minimizes p_{c} elsewhere in an attempt to prevent Ursula from knowing other bits in the oblivious key, in positions unknown to him. As noted in [20], Dave's ability to control p_{c} improves as the angle between the 0-basis and 1-basis, θ, is decreased, making the attack more powerful. However, in both cases (i.e. maximization or minimization of p_{c}), the quantum state Dave sends for this attack lies directly between either pair of ψ or ϕ states shown in Supplementary Figure 1, and thus Ursula will associate a bit value to the measurement that is completely unknown to Dave. Hence, under this attack, Ursula receives a random bit value in response to her query, leading to the cheat sensitive property in [18, 20] (and in our protocol), in which incorrect query results will reveal Dave's dishonest behavior (i.e. over time, Dave will acquire a reputation of providing poor query results).

Furthermore, in our protocol the error correction steps provide additional opportunities for Ursula to verify Dave's honesty, both weakening the above attack as well as providing the possibility of detecting the weakened attack prior to Ursula revealing information about her query. Specifically, the consequence of Dave sending quantum states that minimize p_{c} (in order to prevent Ursula from knowing one or more bits of the oblivious key in random positions) is that Ursula's and Dave's sifted keys are completely uncorrelated (i.e. they have error rates $e_{\mathrm{c}} = e_{\mathrm{i}} = 50\%$). Additionally, since Dave has no knowledge of Ursula's sifted key, the parity bits, $\vec{p}_j$ (see Supplementary Eq. 1), that he sends for error correction will be completely uncorrelated with the parity bits Ursula computes from her measurement results. This allows Ursula to detect a cheating database, and abort the protocol. While this severely restricts Dave's ability to ensure that Ursula does not know bits of the oblivious key in random positions, it does not prevent him from attempting to convince Ursula that she knows a bit in a particular position of his choosing in addition to any bits she learns randomly (in this case, Dave is unsure if Ursula's query corresponds to the position where he conducted the attack, or to an unknown position that Ursula learned randomly). This is because Dave only needs to maximize p_{c} in k bits out of kN bits of the sifted key, which has a negligible effect on the overall error rates for large N. However, this attack has a limited success probability, and if it fails, it may fail in a way that is suspicious to Ursula, again allowing Ursula to abort the protocol (see below for a detailed example). Note that the above verifications occur after the error correction step, but before the shift value is communicated, thus Dave gains no information about Ursula's query if the protocol is aborted.

To illustrate the possibility for Ursula to detect an attempt by Dave to convince her that she knows a particular bit, we consider the parameters discussed above. For $k = 10$ and $\theta = 35.6°$, there is a 37.49% chance that Ursula will believe all k bits are conclusive given this attack. For $k = 9$ and $\theta = 25°$, this probability increases to 64.93%. However, for Dave to convince Ursula that she knows a particular bit of the oblivious key, it is not sufficient for her to believe that all k bits are conclusive, as the error correction procedure must also indicate that her measurement results are correct or correctable (i.e. the error correction procedure results in a error probability $e_{\mathrm{k}} \leq t_{\mathrm{U}}$, where we recall that we have selected $t_{\mathrm{U}} = 10^{-3}$ as the threshold below which Ursula considers a bit to be known). The attack thus becomes more difficult with error correction, since the database must also send parity information to Ursula that is consistent with her measurements. Since Dave's bit values are completely uncorrelated with Ursula's measured bit values, the parity information that Dave sends is essentially random, and Ursula is unlikely to find a low value for e_{k}. In the above examples, Ursula finds $e_{\mathrm{k}} \leq 10^{-3}$ with only 5.92% probability (for $k = 10$ and $\theta = 35.6°$) and 12.73% probability (for $k = 9$ and $\theta = 25°$), showing that this attack has a limited success probability. In addition, the case in which Ursula believes all k bits were measured conclusively is of particular interest as it is very unlikely that she will find a large probability of error in the oblivious key bit after error correction, e_{k}, if the protocol was performed honestly. However, in the above attack, Dave must send parity information that is uncorrelated with Ursula's measurement results, leading to a large amount of uncertainty during Ursula's error correction process and resulting in a high probability of finding a large value for e_{k}. For example, when Ursula believes all k bits were measured conclusively, for $k = 10$ and $\theta = 35.6°$, she expects $e_{\mathrm{k}} \geq 0.15$ with 2.14% probability if Dave is honest, but this value increases to 40.63% given the above attack. For $k = 9$ and $\theta = 25°$, she expects $e_{\mathrm{k}} \geq 0.055$ with 0.71% probability when honest, and 65.63% with the attack. A large value for e_{k} if all k bits are measured conclusively can thus serve as an indication that Dave is attempting to cheat, and allows Ursula to abort the protocol. Furthermore, even if the protocol proceeds and Dave is cheating (e.g. because Dave, by chance, sent consistent parity information), Ursula's and Dave's oblivious key bits after error correction are still uncorrelated, as in the protocol of [18, 20]. This

ensures that the cheat sensitive property of the protocols in [18, 20] discussed above is preserved in our protocol.

Generally speaking, we note that the additional benefits provided by the error correction procedure are relevant to other attack strategies as well. Ursula now has the ability to monitor the aggregate error rates in the system, allowing her to detect any attack by Dave that has a significant effect on the overall error rates. Furthermore, the need for the database to be able to send meaningful parity information during error correction provides an additional hurdle for attacks that cause Dave to lose information about Ursula's measurement results.

B. Database Privacy

On the other hand, a user attacking the protocol seeks to learn as many bits from the database as possible. One method of doing so is to store the photons from Dave in a quantum memory until after he reveals whether he sent a ψ or ϕ state, and then perform an unambiguous state discrimination (USD) measurement [25, 26] to distinguish which of the two remaining states was sent. However, as Dave only reveals information about a quantum state after Ursula has declared that a photon has been detected, every photon that a dishonest Ursula declares as "detected" contributes to her sifted key. As such, any photon that Ursula declares as "detected" but subsequently fails to detect (e.g. because she could not identify when a photon was successfully stored in her quantum memory, or because of loss occurring after the declaration) results in bits in the sifted key of which Ursula has no knowledge. Successfully performing an USD attack thus requires a heralding signal indicating that a photon was successfully stored in the quantum memory, and the ability to recall the photon from the quantum memory with near 100% efficiency. For the following analysis, we assume a heralding signal in conjunction with a perfect quantum memory (i.e. one that introduces no error into the quantum states, and has 100% efficiency; a realistic quantum memory, such as those assumed in the noisy-storage model, would reduce the effectiveness of the attack), and that there are no other sources of loss that reduce the success probability of the USD measurement.

If Ursula is able to perform an USD measurement, this allows her to maximize the probability that the quantum measurements will give conclusive results. As shown in [18], the probability of conclusive results increases only slightly when performing USD measurements, resulting in the user only learning a few more bits than when making honest measurements. Furthermore, the advantage decreases as θ is decreased [20]. Additionally, in the presence of error correction, the advantage of performing an USD measurement further decreases. This is because the USD measurement gains no information from inconclusive results, essentially exchanging this information for an increased probability of obtaining a conclusive result. However, the partial information from inconclusive results is useful during error correction, and can even allow Ursula to know the value of the oblivious key bit in some instances in which not all measurements were conclusive. As such, error correction can reduce the effectiveness of the USD attack. Performing USD measurements when using the code with $k = 10$ and $\theta = 35.6^\circ$ only increases the average number of bits the user knows from $\bar{n} = 3.89$ to $\bar{n} = 11.15$ — a rather small gain for a database of 10^6 bits. For the code using $k = 9$ and $\theta = 25^\circ$, performing USD measurements actually decreases the average number of bits the user knows from $\bar{n} = 4.35$ to $\bar{n} = 1.00$. This decrease is due to the fact that at this smaller value of θ, the value of the partial information gained from inconclusive measurements outweighs the slightly improved probability for a conclusive measurement offered by the USD measurement. Note that these results are based on having the same error rate as for the honest measurements, which may not be a realistic assumption given that a different measurement apparatus is required. The issue of error rates differing from those used to select the error-correcting code is addressed separately below so as to isolate this effect from that of the USD measurement.

C. Error rate estimation

Finally, since Ursula and Dave have an adversarial nature in the private query protocol, accurately characterizing the error rate in the system in order to select an error-correcting code is not straightforward. In particular, Ursula would like the database to believe that the error rate is higher than in reality, as Dave would then select an error-correcting code that gives her more information, allowing her to learn more bits from the database. To avoid this problem, Dave can determine the amount of information a user will learn from the protocol based solely on the error introduced by devices directly under his control. In fact, he can even choose to deliberately introduce additional noise in order to provide the desired level of database security. Additional imperfections in the system would cause the user to experience a higher error rate than Dave's estimate, leading to her learning fewer bits than the database predicts. To show that there is a regime that allows the protocol to succeed from the user's perspective while still providing good database security, we re-examine the error-correcting codes that we have considered thus far using the

Supplementary Table I: Comparison of simulation results for a user experiencing higher error rates than those used by Dave to select an error-correcting code. The columns labeled “all” show experimental results obtained using standard detectors ($\theta = 35.6°$, $k = 10$), or simulation results with improved detectors ($\theta = 25°$, $k = 9$), as taken from Tables 2 and 3 of the main text, and represent the actual results of the protocol as influenced by noise due to all imperfections. The columns labeled “source only” represent Dave's predicted results for the protocol, based on an error rate estimation considering only noise introduced by his source.

	$\theta = 35.6°$, $k = 10$		$\theta = 25°$, $k = 9$	
noise	all	source only	all	source only
p_c (%)	16.1	15.9	9.22	9.14
e_c (%)	4.4	2.5	1.91	1.38
e_i (%)	41.24	40.89	45.12	45.11
$\bar{n}$ (bits)	3.89	14.32	4.35	10.67
$\bar{m}$ (%)	6.03	6.69	0.96	0.93

parameters shown in the columns labeled “source only” in Supplementary Table I, where noise in the system has been reduced compared to the original parameters in the main text (shown in the columns labeled “all”). Note that the effect of the lower noise observed by the database is not just a lower error rate in the conclusive measurements, e_c, in the “source only” columns — the other parameters are affected as well. The error rate for inconclusive measurements, e_i, is affected by the same noise sources as e_c, but the effect on e_i is smaller as the error for inconclusive measurements is dominated by uncertainty inherent in the quantum measurement. Hence, e_i in the “source only” columns is only slightly lower than in the “user” columns. The total number of conclusive results is reduced slightly as the number of conclusive results recorded due to noise events is lower. Hence, the probability of conclusive measurements, p_c, is lowered slightly in the “source only” columns. Supplementary Table I also shows the results for the average number of bits learned by the user, $\bar{n}$, and the average proportion of the database for whic Dave considers Ursula to have significant partial information, $\bar{m}$, for the original parameters in the “user” columns, as well as for a lower error rate that can be used to select the error-correcting code in the “source only” columns. As can be seen, the reduction in error rates does not result in a large increase in the potential amount of information gained by a user who experiences no additional error. Thus, it is possible for an error-correcting code that is selected based on local error rates to both provide the database with good security and allow the protocol to be successful for a user experiencing higher error rates.

[1] Lo, H.-K. Insecurity of quantum secure computations. *Phys. Rev. A* **56**, 1154–1162 (1997).

[2] Naor, M. & Pinkas, B. Distributed oblivious transfer. In *Proc. 6th Int. Conf. on the Theory and Applicat. of Cryptology and Inf. Security: Advances in Cryptology*, ASIACRYPT '00, 205–219 (2000).

[3] Blundo, C., D'Arco, P., De Santis, A. & Stinson, D. On unconditionally secure distributed oblivious transfer. *J. Cryptol.* **20**, 323–373 (2007).

[4] Rabin, M. O. How to exchange secrets by oblivious transfer. Tech. Rep., Harvard University (1981).

[5] Kleinjung, T. *et al.* Factorization of a 768-bit RSA modulus. In *Proc. 30th annual conf. on Advances in cryptology*, CRYPTO'10, 333–350 (2010).

[6] Shor, P. W. Polynomial-time algorithms for prime factorization and discrete logarithms on a quantum computer. *SIAM J. Comput.* **26**, 1484–1509 (1997).

[7] König, R., Wehner, S. & Wullschleger, J. Unconditional security from noisy quantum storage. *IEEE Trans. Inf. Theory* **58**, 1962 –1984 (2012).

[8] Schaffner, C. Simple protocols for oblivious transfer and secure identification in the noisy-quantum-storage model. *Phys. Rev. A* **82**, 032308 (2010).

[9] Erven, C. *et al.* An experimental implementation of oblivious transfer in the noisy storage model. *arXiv:1308.5098* (2013).

[10] Lvovsky, A. I., Sanders, B. C. & Tittel, W. Optical quantum memory. *Nat. Photon.* **3**, 706–714 (2009).

[11] Tittel, W. *et al.* Photon-echo quantum memory in solid state systems. *Laser Photonics Rev.* **4**, 244–267 (2010).

[12] Hammerer, K., Sørensen, A. S. & Polzik, E. S. Quantum interface between light and atomic ensembles. *Rev. Mod. Phys.* **82**, 1041–1093 (2010).

[13] Simon, C. *et al.* Quantum memories. *Eur. Phys. J. D* **58**, 1–22 (2010).

[14] Schindler, P. *et al.* Experimental repetitive quantum error correction. *Science* **332**, 1059–1061 (2011).

[15] Bussières, F. *et al.* Prospective applications of optical quantum memories. *arXiv:1306.6904* (2013).

[16] Giovannetti, V., Lloyd, S. & Maccone, L. Quantum private queries. *Phys. Rev. Lett.* **100**, 230502 (2008).

[17] De Martini, F. *et al.* Experimental quantum private queries with linear optics. *Phys. Rev. A* **80**, 010302 (2009).

[18] Jakobi, M. *et al.* Practical private database queries based on a quantum-key-distribution protocol. *Phys. Rev. A* **83**, 022301 (2011).

[19] Scarani, V., Acín, A., Ribordy, G. & Gisin, N. Quantum cryptography protocols robust against photon number splitting attacks for weak laser pulse implementations. *Phys. Rev. Lett.* **92**, 057901 (2004).

[20] Gao, F., Liu, B., Wen, Q.-Y. & Chen, H. Flexible quantum private queries based on quantum key distribution. *Opt. Express* **20**, 17411–17420 (2012).

[21] Hwang, W.-Y. Quantum key distribution with high loss: Toward global secure communication. *Phys. Rev. Lett.* **91**, 057901 (2003).

[22] Wang, X.-B. Beating the photon-number-splitting attack in practical quantum cryptography. *Phys. Rev. Lett.* **94**, 230503 (2005).

[23] Ma, X., Qi, B., Zhao, Y. & Lo, H.-K. Practical decoy state for quantum key distribution. *Phys. Rev. A* **72**, 012326 (2005).

[24] Wehner, S., Curty, M., Schaffner, C. & Lo, H.-K. Implementation of two-party protocols in the noisy-storage model. *Phys. Rev. A* **81**, 052336 (2010).

[25] Herzog, U. & Bergou, J. A. Optimum unambiguous discrimination of two mixed quantum states. *Phys. Rev. A* **71**, 050301 (2005).

[26] Raynal, P. Unambiguous state discrimination of two density matrices in quantum information theory. *arXiv:quant-ph/0611133v1* (2006).
